# Lactate-Induced Glucose Output Is Unchanged by Metformin at a Therapeutic Concentration – A Mass Spectrometry Imaging Study of the Perfused Rat Liver

**DOI:** 10.3389/fphar.2018.00141

**Published:** 2018-02-22

**Authors:** Giulio Calza, Elisabeth Nyberg, Matias Mäkinen, Rabah Soliymani, Annunziata Cascone, Dan Lindholm, Emanuele Barborini, Marc Baumann, Maciej Lalowski, Ove Eriksson

**Affiliations:** ^1^Meilahti Clinical Proteomics Core Facility, Helsinki Institute of Life Science, Faculty of Medicine, University of Helsinki, Helsinki, Finland; ^2^Department of Biochemistry and Developmental Biology, Faculty of Medicine, University of Helsinki, Helsinki, Finland; ^3^Minerva Foundation Institute for Medical Research, Helsinki, Finland; ^4^Tethis S.p.A., Milan, Italy

**Keywords:** metformin, diabetes, mass spectrometry imaging, liver, gluconeogenesis, lactate

## Abstract

Metformin is the first line drug for type 2 diabetes but its molecular mechanisms remain unclear. Here, we have studied the acute effect of a therapeutically relevant intrahepatic concentration of metformin on glucose production from lactate. We selected the perfused rat liver as experimental system since it enables the complete control of drug dosage. We used MALDI (matrix-assisted laser desorption/ionization) mass spectrometry imaging to estimate the concentration of metformin in the livers and we measured the concentration of glucose in the effluent medium under basal conditions and following lactate addition. MALDI mass spectra of thin-sections of freeze-clamped rat liver perfused with metformin showed a peak at 130.16 *m/z* which was unambiguously assigned to metformin. The mass spectrometric detection limit was at a tissue concentration of about 250 nM, and uptake of metformin from the perfusion medium to the liver occurred with a K_m_ of 0.44 mM. Metformin was evenly distributed in the liver irrespective of its concentration in the perfusion medium and the duration of a perfusion. At a parenchymal concentration of 30 μM, metformin did not induce any significant suppression of the basal or lactate-induced glucose release from the liver. These results show that matrix-assisted laser desorption/ionization mass spectrometry imaging can be applied to estimate the tissue concentration and distribution of metformin in a therapeutically relevant micromolar concentration range. Our findings challenge the view that metformin causes an inhibition of glucose release from the liver by an acute inhibition of mitochondrial glycerol 3-phosphate dehydrogenase (EC 1.1.5.3).

## Introduction

Metformin is a hydrophilic biguanide (1,1-dimethylbiguanide) compound currently prescribed to more than 100 million persons for the treatment of type 2 diabetes (T2D) (Maruthur et al., 2016). Metformin is also used in comorbidities of T2D including polycystic ovary syndrome (Patel and Shah, 2017) and non-alcoholic fatty liver disease (Rouabhia et al., 2014). T2D is associated with an increased risk of both Alzheimer’s disease and vascular dementia (Biessels et al., 2006), and some studies indicate that metformin may have a direct neuroprotective effect (Patrone et al., 2014). Patients under treatment with metformin for T2D have a decreased mortality from cancer as revealed by several retrospective pharmaco-epidemic studies (Evans et al., 2005; Currie et al., 2009; Libby et al., 2009; Zhang et al., 2012). These encouraging discoveries have generated much interest in the repositioning of metformin for use in diseases other than T2D.

Metformin is highly water soluble and its uptake into tissues is mediated by the organic cation transporters OCR1/SLC22A1, OCR2/SLC22A2, the multidrug and toxin extrusion transporters MATE1/SLC47A1 and MATE2/SLC47A2, and the plasma membrane monoamine transporter (Christensen et al., 2011). Oral metformin administration at a therapeutic dose in humans results in a maximal plasma concentration of about 10 μM (Tucker et al., 1981; Christensen et al., 2011). In rodents, metformin has been shown to accumulate into the gut, liver, kidney, and skeletal muscle (Wilcock and Bailey, 1994; Quinn et al., 2013), while less is known about the uptake into the CNS and its various regions. Metformin can penetrate into certain tumors, and concentrations reaching 100 μM have been measured in xenograft-bearing mice following oral administration (Chandel et al., 2016; Dowling et al., 2016).

Several biochemical effects of metformin have been studied in detail including (i) an inhibition of complex I of the mitochondrial respiratory chain (El-Mir et al., 2000; Owen et al., 2000; Bridges et al., 2014), (ii) an activation of AMP-dependent protein kinase (Zhou et al., 2001), (iii) a decrease in cAMP with an attenuated activation of PKA (Miller et al., 2013), and (iv) an inhibition of mitochondrial glycerol 3-phosphate dehydrogenase (Madiraju et al., 2014). However, due to the broad range of concentrations employed in the biochemical studies, frequently 10–100 times higher than that measured in the plasma of patients (He and Wondisford, 2015), it is challenging to establish causal links between the biochemical effects of metformin and its antidiabetic activity, thought to result mainly from a reduction in the glucose release from the liver (Foretz et al., 2014).

Furthermore, varying effects of metformin on glucose release have been reported, depending on study design. During ongoing metformin therapy, a reduction in the glucose release from the liver can be observed (Jackson et al., 1987; Wu et al., 1990; Perriello et al., 1994; Hundal et al., 2000), while acute administration of metformin does not exert any effect on glucose release (Sum et al., 1992; Widén et al., 1992). In contrast, perfusion with metformin at a supra-therapeutic concentration causes an acute reduction in the glucose production from lactate in rodent liver (Radziuk et al., 1997). It is evident that several processes influence the onset of the glucose-lowering effect, including the uptake into the hepatocytes, determined by plasma membrane transporters, and the response of the cellular machinery, requiring time for signal transduction events and transcriptional activation to take place. To clarify the molecular mechanisms of metformin, it is necessary to control these processes in the experimental system employed.

This study was performed in order to determine the acute effect of a therapeutic concentration of metformin on liver glucose release, employing the perfused rat liver as an experimental system. We utilized MALDI-MSI (Vaysse et al., 2017) to determine the uptake and distribution of metformin into the parenchyma of the liver and we devised conditions for loading the liver with metformin. We showed that metformin can be detected at sub-micromolar concentrations in cryo-sections of freeze-clamped liver using MALDI-MSI, and we determined the kinetic constants of metformin uptake into the liver. We assessed the acute effect of metformin on both the basal and the lactate-induced glucose release, and our findings challenge the view that the antidiabetic effect of metformin is due to inhibition of gluconeogensis from lactate through a direct effect on glycerol 3-phosphate dehydrogenase (Madiraju et al., 2014). This study demonstrates the power of MALDI-MSI for assessing the tissue concentration and distribution of metformin, and the technique will be useful for future studies of metformin in T2D, cancer and neurodegenerative states.

## Materials and Methods

### Animals and Liver Perfusions

**FIGURE 1:**
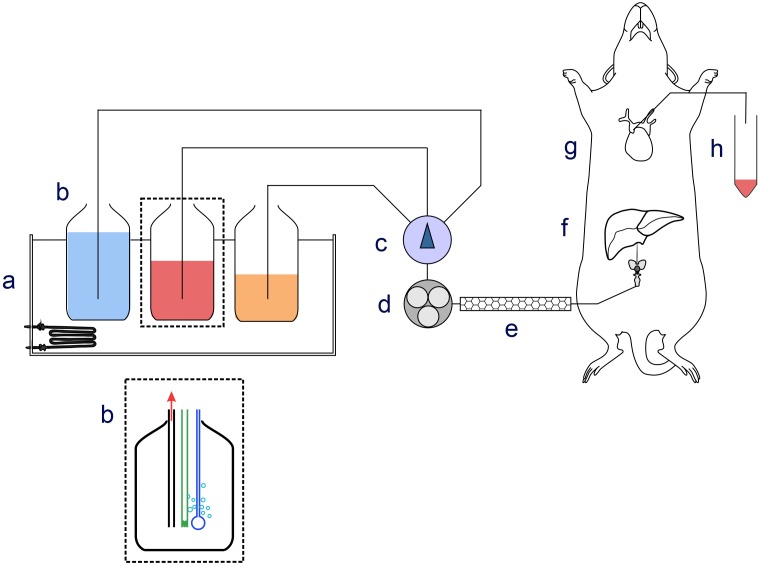
Schematic drawing of the perfusion apparatus. Components of the perfusion apparatus: (a) thermostatic bath; (b) bottles containing perfusion medium equilibrated with O_2_/CO_2_ (19:1) at pH 7.40 ± 0.05; (c) manifold; (d) peristaltic pump; (e) thermostat at 37 ± 1°C; (f) cannula for ingoing medium; (g) cannula inserted in lower vena cava collecting outgoing medium; (h) and fraction collector.

This study was carried out in accordance with the recommendations of the Animal (Scientific Procedures) Act 1986 of the United Kingdom Parliament, Directive 2010/63/EU of the European Parliament and the Guide for the Care and Use of Laboratory Animals published by the United States National Institutes of Health (NHI Publication No. 85-23, revised 1996). The ethical permit for use of laboratory rats and the protocol was approved by the Helsinki University Laboratory Animal Center (permit No. KEK18-016). Male Wistar rats (Envigo, Netherlands) weighing 140–160 g were starved for 24 h prior to experiments and anesthetized by intraperitoneal injection of sodium thiopental (60 mg/kg) (Eriksson et al., 1999). The abdomen of the rat was opened and heparin (100 IU/kg) was injected into the inferior vena cava. After 2 min a 24G cannula was inserted into the portal vein and the liver was perfused with perfusion medium composed of glucose-free Hank’s balanced salt medium (HBSS) supplemented with 1.2 mM Ca^2+^, 0.9 mM Mg^2+^, 0.5 mM pyruvate, 0.5 mM glutamine, 0.5 mM acetate, 10 mM Hepes, and 1 μM glucagon when indicated. The medium was equilibrated with O_2_/CO_2_ (19:1) at pH 7.40 ± 0.05, and the temperature was 37 ± 1°C (for a scheme of the perfusion apparatus see **Figure 1**). The portal vein branches going to the median, left, and caudate lobes of the liver were occluded by ligatures, and these lobes were removed, leaving the right lateral lobe intact. The flow was 5 ml/(min × gram wet liver weight). An 18G cannula was inserted into the vena cava through the right atrium of the heart, in order to collect the effluent medium from the liver in 1 min fractions for glucose determination. The preparation took less than 10 min from opening the abdomen of the rat. Following a stabilization period of 5 min, perfusions were performed according to the experimental protocols detailed below. After the perfusion experiment, the liver right lateral lobe was excised and freeze-clamped with liquid nitrogen.

### Perfusion Protocols

**FIGURE 2:**
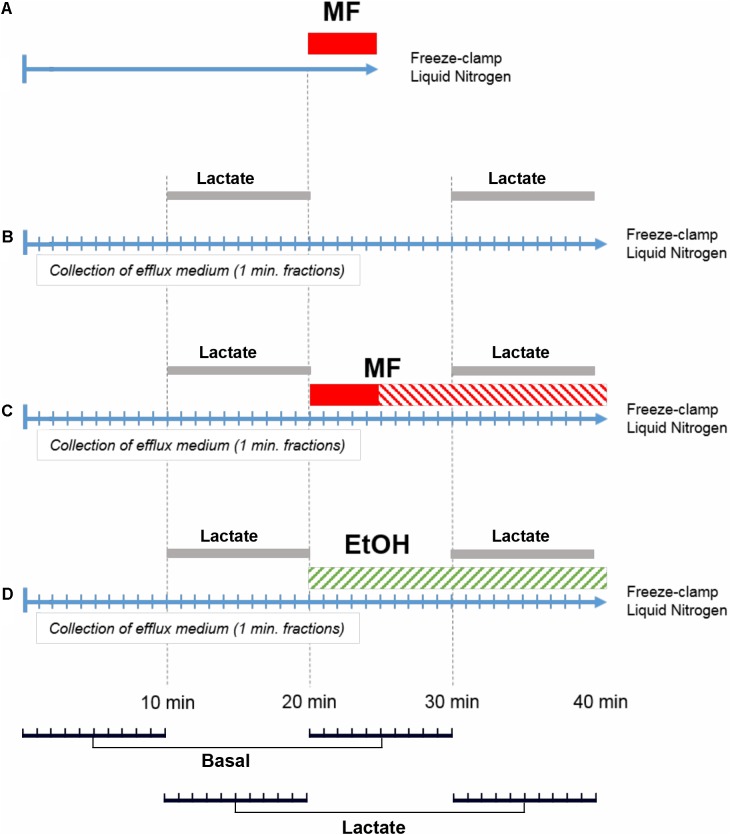
Perfusion protocols. Perfusions according to protocol **(A)** were performed to measure the intrahepatic concentration of metformin and to optimize the concentration of metformin in the perfusion medium during the loading period. Perfusions according to protocol **(B)** were control experiments with two periods of D-lactate (5 mM) challenge (*Lactate*). Perfusions according to protocol **(C)** performed to determine the effect of metformin (*MF*) on glucose release and its response to D-lactate. Loading concentration (400 μM) with metformin is indicated in red, and steady state concentration of metformin (40 μM) is indicated by red stripes. Perfusions according to protocol **(D)** were performed with 25 mM ethanol (*EtOH*). For statistical analysis, paired comparisons were made for the efflux glucose concentration at t = 1 and 21 min, t = 2 and 22 min, and similarly until t = 20 and 40 min, resulting in 10 pairs for basal conditions (*Basal*) and 10 pairs for lactate-induced conditions (*Lactate*), as indicated in the bottom panel.

Four different perfusion protocols were performed as shown schematically in **Figure 2**. The assignment of rats to different groups was randomized. Protocol A served to determine the concentration of metformin in the perfusion medium required to reach a parenchymal concentration of 30 μM in 5 min. Protocols B to D were used to determine the effect of metformin or ethanol on the basal and lactate-induced glucose release. Time t = 0 indicates the end of the stabilization period as described above. In protocol A, livers were perfused with perfusion medium without metformin for 20 min, followed by 5 min with perfusion medium supplemented with 100, 250, 500 μM, 1 or 2.5 mM metformin, followed by a brief washout for 20 s with perfusion medium without metformin. The liver lobe was then resected, immediately freeze-clamped in liquid nitrogen at -196°C, and used for analysis by MALDI-MSI. In protocol B, which was the control, livers were perfused for 10 min with perfusion medium, followed by 10 min with perfusion medium supplemented with 5 mM D-lactate. This was followed by 10 min with perfusion medium and 10 min with perfusion medium supplemented with 5 mM D-lactate, after which the liver was freeze clamped. In protocol C, livers were perfused for 10 min with perfusion medium. At 10 min, the perfusion medium was supplemented with 5 mM D-lactate. At 20 min, the D-lactate was omitted and the livers were perfused for 5 min with perfusion medium supplemented with 400 μM metformin. At 25 min, livers were perfused for another 5 min with perfusion medium supplemented with 40 μM metformin until the end of the perfusion. At 30 min, the perfusion medium was supplemented with 5 mM D-lactate, and at 40 min the D-lactate was omitted. In protocol D, livers were perfused for 10 min with perfusion medium followed by 10 min with perfusion medium supplemented with 5 mM D-lactate. At 20 min, livers were perfused for 10 min with perfusion medium supplemented with 25 mM ethanol, followed at 30 min by 10 min with perfusion medium supplemented with 5 mM D-lactate and 25 mM ethanol. By performing the transition from basal to lactate-induced glucose production twice in each perfusion, every liver served as its own control. This procedure reduced errors due to differences in the liver lobe size, and variations in the liver metabolism between the different animals.

### Cryo-sections

The freeze-clamped right lateral lobe was cut into 12 μm thick transversal sections using a Leica CM3050 cryostat (Leica Microsystems GmbH, Germany), and thaw-mounted onto indium tin oxide (ITO)-coated MALDI target slides (Bruker Daltonics, BD, Germany), covered with a nano-structured layer of TiO_2_ (Tethis S.p.A., Milan, Italy), which improves tissue adhesion during the MSI process. Prior to matrix deposition target slides were dried for 10 min in a SpeedVac SVC200H (Savant, Farmingdale, NY, United States), and optical images of the tissues were acquired using a flat-bed scanner (Epson Perfection V750 Pro) at a resolution of 2400 px/inch.

### Deposition of Matrix Substances for MALDI Mass Spectrometry

A solution of 7 mg/ml of HCCA in 50% acetonitrile/0.1% trifluoroacetic acid was deposited onto the liver thin-section using an iMatrixSpray instrument (Tardo Gmbh, Subingen, Switzerland), applying 10 cycles of deposition, resulting in a homogeneous coating (0.57 ± 0.05 μg/cm^2^) of dry HCCA.

### MALDI Mass Spectrometry

Liver thin-sections were scanned with a 150 μm raster on a Bruker UltrafleXtreme 2 kHz MALDI-TOF/TOF Mass Spectrometer in Reflectron positive mode, acquiring in the 20–500 Da mass range and summing 2000 laser shots per raster position. For analyses of the fragmentation of the parent ion measurements were made in LIFT MS/MS mode. The resulting FlexImaging 4.1 (Bruker Daltonics, Bremen, Germany) files, containing the acquired spectra from the measurement regions, were imported into SCiLS Lab 2017a software (^1^Bremen, Germany) for subsequent Total ion current (TIC) normalization, peak alignment, and data visualization.

### Quantification of the Mass Spectrometric Signal of Metformin

To estimate the concentration of metformin in perfused livers two methods were used to generate a standard curve. In the standard under the tissue method, known quantities of metformin were dissolved in water and spotted directly onto the ITO slides. The applied metformin solution was allowed to dry, and the metformin-containing spots were marked. Liver thin-sections from control livers which had been perfused without metformin where then placed over of the metformin spots. In the standard over the tissue method, known quantities of metformin dissolved in water were applied on top of mounted liver thin-section. The applied metformin solution was allowed to dry and the areas occupied by the dried metformin-containing spots were marked before matrix deposition. Standard curves were generated by the standard under, and standard on top of the thin-section methods, using the median intensity of the metformin signal in each spot region. For calculating the parenchymal metformin concentration, the mean values of the standard under and standard on top of the thin-section methods were used. Signal-to-noise ratio was calculated using FlexAnalysis version 3.4.

### Glucose Determination

The glucose concentration of the effluent medium was measured as NADP reduction at λ = 340 nm using a coupled enzyme assay. The reaction mixture contained 1 mM ATP, 2.5 mM NADP, hexokinase (0.1 U), and glucose 6-phosphate dehydrogenase (0.1 U) dissolved in HBBS medium supplemented with 1.2 mM Ca^2+^, and 0.9 mM Mg^2+^. The reaction was allowed to proceed for 30 min at room temperature after the addition of the enzyme mixture. Absorbance was measured using an EnSpire Multimode plate reader (Perkin Elmer). The average concentration of glucose in the effluent medium during the first 10 min of the perfusion was normalized to one, because the size of the right lateral lobe varied between different animals. The glucose release during the first 10 min of the perfusion was 64.9 ± 18 μM (mean ± SD, *n* = 30 for all experimental groups together). The person performing the glucose analyses had no knowledge about the experimental group of the samples.

### Statistical Analysis

The data and statistical analysis comply with the recommendations on experimental design and analysis in pharmacology (Curtis et al., 2015). For the MALDI-MSI data spectra were normalized with the TIC method (Deininger et al., 2011) which normalizes every spectrum separately by dividing each spectrum intensity by the sum of all its intensities. Peaks were aligned to local maxima (Alexandrov and Kobarg, 2011) by setting the minimal interval width to 0.125 *m/z*. Baseline removal was not performed.

Data from glucose determinations were collected in Excel (Microsoft) and statistical analysis was performed using the IBM SPSS Statistics 24.0 software (IBM Corporation, Armonk, NY, United States). The raw data were analyzed independently by two of the co-authors to ensure correctness of the conclusions. To test for statistically significant differences in glucose release, pairwise comparisons were made of the effluent glucose concentration at t = 1 min with t = 21, t = 2 min with t = 22 min, and similarly until t = 20 min which was compared with t = 40 min, for each individual rat liver. For each perfusion, 20 measurement pairs were hence formed, of which the 10 pairs where for basal glucose release and 10 pairs for lactate-induced glucose release, as indicated in **Figure 2**, lower panel. Testing for normal distribution was performed using the Shapiro–Wilk test, and for statistical significance using Student’s *t*-test under the null hypothesis that there was no difference in glucose concentration before and after addition of metformin or ethanol, respectively. A *p* < 0.01 was considered significant and this was marked with an asterisk in the graph.

### Chemicals and Reagents

Metformin was from Sigma-Aldrich (pharmaceutical secondary standard grade, Lot#LRAA8975). HCCA was of mass spectrometry grade form Sigma-Aldrich. All other chemicals were of highest grade and purchased from Sigma-Aldrich.

## Results

### Mass Spectrometric Properties of Metformin

**FIGURE 3:**
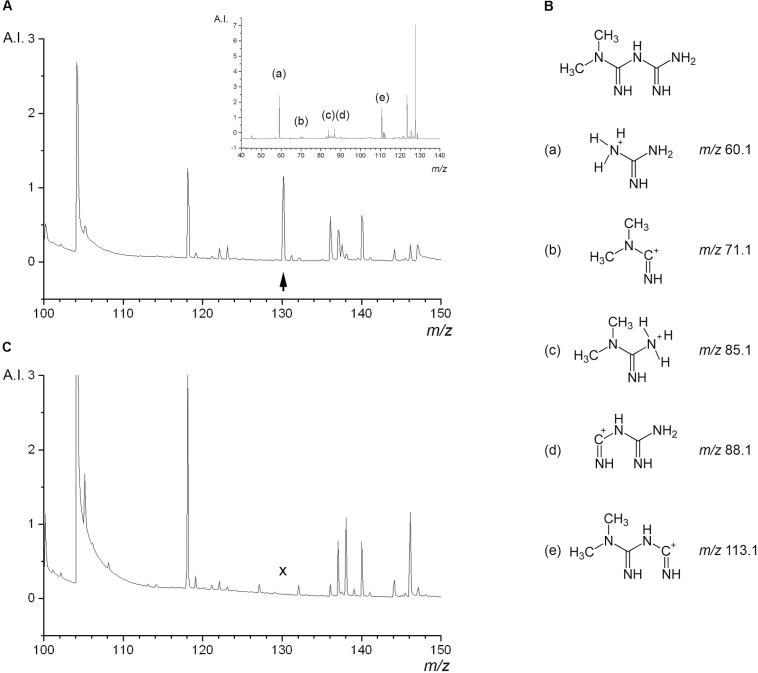
Mass spectrometric properties of metformin in liver tissue background. Thin-sections of freeze-clamped rat livers perfused with or without metformin were analyzed by MALDI TOF mass spectrometry. (**A**, main image) Shows the mass spectrum of liver tissue containing metformin, the peak at 130.16 *m/z* corresponding to the parent molecular mass of metformin is indicated with an *arrowhead*. (**A**, inset) Shows the LIFT MS/MS spectrum of the 130.16 *m/z* parent ion with the peaks of fragment ions of metformin. **(B)** Structure of metformin and detected fragment ions indicated with letters. **(C)** Shows the mass spectrum of liver tissue perfused without metformin, lacking the peak at 130.16 *m/z* (*cross*). Arbitrary intensity (A.I.) represents the unit reported by the FlexImaging^TM^ software package.

In order to select the best condition for the ionization of metformin we tested several different MALDI matrix substances on the metformin-containing liver thin-sections, including HCCA, 2-picolinic acid, ferulic acid, sinapic acid, and 2,5-dihydroxy benzoic acid. This revealed that HCCA yielded the best results in terms of peak shape and intensity (data not shown). We first characterized the MALDI TOF mass spectra of thin-sections from rat livers that had been perfused with 500 μM metformin according to protocol A (**Figure 3A**). A distinct peak at 130.16 *m/z* corresponding to the molecular weight of metformin was observed specifically in thin-sections of metformin-treated liver. To validate the assignment of the 130.16 *m/z* peak we performed MS/MS analyses using LIFT fragmentation (**Figure 3A**, inset), which yielded a peak pattern consistent with the theoretical and measured fragmentation of metformin (MassBank data base, entry KO003376), and literature data (Michel et al., 2015). Five ions could be detected, corresponding to the fragment ions of metformin (**Figure 3B**). The control liver thin-sections did not yield any fragmentation spectrum due to the absence of detectable precursor ions at 130.16 *m/z* (**Figure 3C**). These findings confirmed that the 130.16 *m/z* peak was metformin.

### Estimation of the Parenchymal Metformin Concentration

**FIGURE 4:**
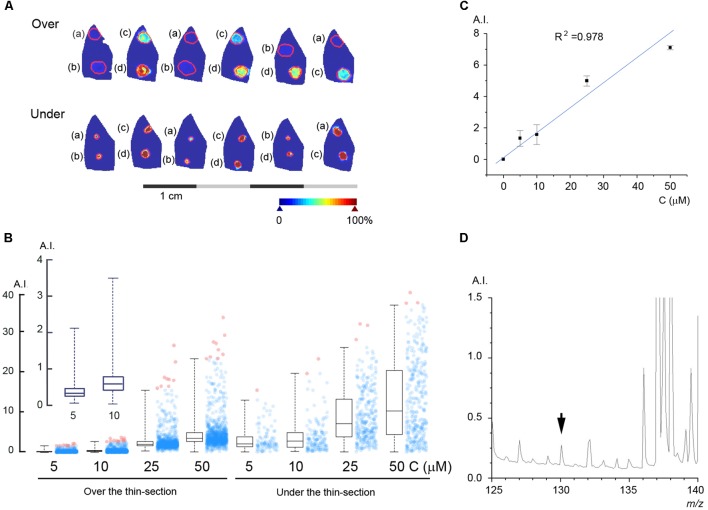
Estimation of the metformin concentration in the liver parenchyma. **(A)** Known quantities of metformin were spotted either on top of (*Over*) or under (*Under*) thin-sections of rat livers perfused without metformin: (a) 5 μM, (b) 10 μM, (c) 25 μM, and (d) 50 μM. **(B)** Box plot of the pixel values obtained for each spot of metformin. Box lines are median, first and third quartiles, and whiskers show the 2^nd^ and 98^th^ percentiles. The average number of pixels for each ROI was 896. (**B**, inset) Shows the peak intensity for 5 and 10 μM metformin. **(C)** Standard curve with linear regression calculated from the average of the values obtained by applying standard on top of and under the thin-section, respectively. **(D)** The mass spectrum of a liver thin-section with 250 nM metformin, as indicated (*arrowhead*). Arbitrary intensity (A.I.) is reported as in **Figure 3**.

In order to estimate the concentration of metformin in the perfused liver we used two different methods, standard under and standard on top of the thin-section as described in Section “Materials and Methods.” The results of these experiments are shown in **Figure 4A**, where known quantities of metformin were spotted either under or above thin-sections of rat liver perfused according to protocol A without metformin. The intensity of the peak at 130.16 *m/z* is shown in pseudo-coloration, and the borders of the regions of interest (ROI) of the thin-sections containing metformin are marked in red. The intensity of the peak at 130.16 *m/z* was plotted for each pixel in the ROIs and the resulting histograms with the distribution in pixel values are shown in **Figure 4B**. The standard curve was constructed by calculating the average of the respective median value obtained for the standard under and standard on top of the thin-section methods (**Figure 4C**), in order to simulate the condition of metformin being embedded within the tissue matrix. To estimate the mass spectrometric detection limit of metformin in the liver, we incrementally decreased the metformin concentration, and the results yielded that, at a concentration of 250 nM, the metformin peak at 130.16 *m/z* could be detected at a signal-to-noise ratio of three (**Figure 4D**).

### Uptake of Metformin by the Perfused Liver

**FIGURE 5:**
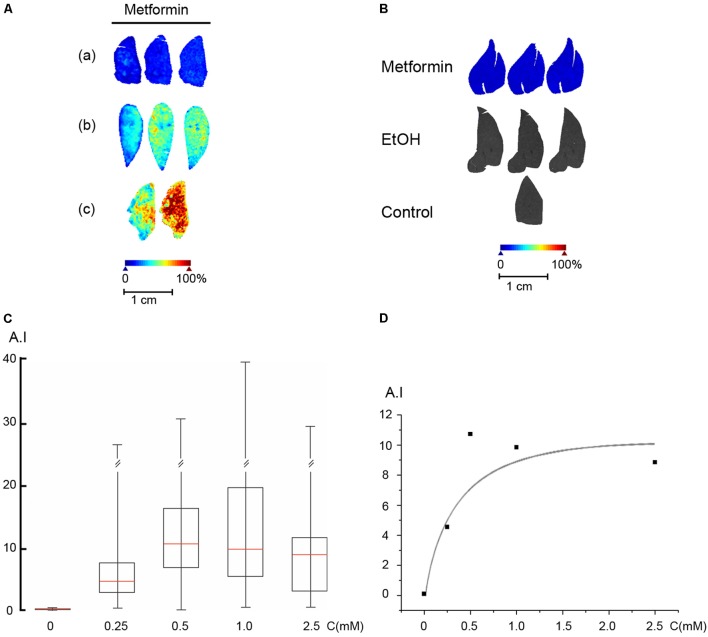
Uptake of metformin into the perfused liver. **(A)** Rat livers were perfused according to protocol A with metformin in the perfusion medium, (a) 250 μM, (b) 500 μM, and (c) 1 mM. **(B)** Rat livers were perfused according to protocol C (*Metformin*), protocol D (*EtOH*), or protocol B (Control), and freeze-clamped the end of the perfusion. The metformin concentration was 30 ± 10 μM (*n* = 5). **(C)** Rat livers were perfused according to protocol A. Box plot of the signal intensity of metformin versus the concentration of metformin used in the perfusion medium. Box plot parameters were as in **Figure 4**. **(D)** Plot of the signal intensity versus metformin concentration. A double reciprocal plot (not shown) yielded a K_m_ of 0.44 mM. Arbitrary intensity (A.I.) is reported as in **Figure 3**.

We first established the concentration of metformin required in the perfusion medium to reach a concentration of 30 μM in the liver parenchyma, using perfusion protocol A. The perfusion medium was supplemented with metformin at concentrations ranging from 100 μM to 5 mM for 5 min, followed by a brief washout with perfusion medium lacking metformin. Liver lobes were freeze-clamped and analyzed by MALDI-MSI, and the parenchymal metformin concentration was assessed using the established standard curve (**Figure 5A**). For the perfused livers, we used the median values of the peak at 130.16 *m/z* for all pixels from the whole area of the liver thin-section. As shown (**Figure 5A**), metformin was evenly distributed in the liver lobe with local variations in the range of less than 20%. We then analyzed the intrahepatic metformin concentration as a function of the metformin concentration used in the perfusion medium according to protocol A. These results indicated that metformin was effectively accumulated into the liver parenchyma with an estimated K_m_ of 0.44 mM and a V_max_ of 0.2 μmol/(gxmin) (**Figures 5C,D**). Based on these results, we selected to use 400 μM metformin for the loading period, and 40 μM for the steady state in the perfusions according to protocol C. To ascertain that the concentration of metformin remained constant until the end of the experiment (protocol C), we freeze-clamped livers at the end of the perfusion and determined their metformin content. The results of these experiments (**Figure 5B**), revealed that the concentration of metformin remained stable at about 30 μM during the course of the experiment. As anticipated, there was no detectable signal above baseline at 130.16 *m/z* in control livers in the absence of metformin, or in livers perfused with ethanol (**Figure 5B**).

### Effect of Metformin on Glucose Release

**FIGURE 6:**
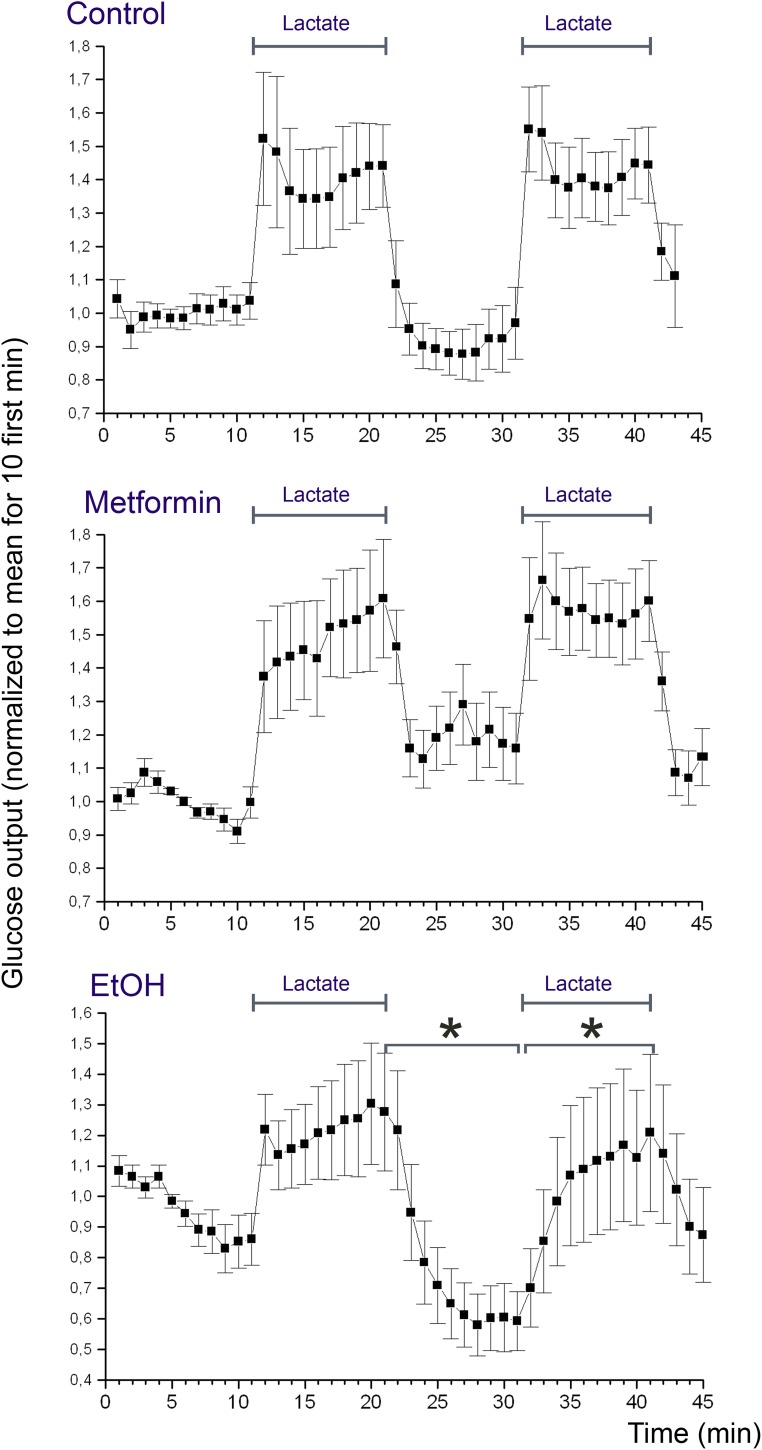
Glucose release from the perfused liver. Rat livers were perfused according to protocol B (*Control*), C (*Metformin*), or D (*EtOH*). The presence of lactate in the medium is indicated with bars (*Lactate*). The effluent medium was collected in 1 min fractions following passage through the liver. The glucose concentration of the effluent medium was determined using a coupled enzyme assay (mean values ± SD, *n* = 10 for each experimental group). Concentrations were normalized by setting the average of the 10 first measurement points to one. For each rat, the glucose concentration of the effluent medium after addition of metformin or ethanol was compared to that before addition. Statistical significance (*p* < 0.01) was assessed as described in Section “Statistical Analysis.”

Rats were starved 24 h prior to experiments, to deplete the liver from glycogen. Livers were perfused according to protocol B using 5 mM D-lactate to promote glucose release. **Figure 6** shows the concentration of glucose in the efflux medium during the course of the perfusion. It can be appreciated that addition of lactate induced a marked increase in the glucose release of about 1.5-fold over the basal level, and this increase was quantitatively similar during the first and second period with lactate (**Figure 6**, Control). In the next series of perfusions (protocol C), we supplemented the perfusion medium with metformin starting at t = 20 min in order to study the effect of metformin on the glucose release. The result of this experiment revealed that metformin did not induce any significant suppression neither in the basal glucose release nor in the lactate-induced increase in glucose release (**Figure 6**, Metformin). In the positive controls (protocol D), we supplemented the perfusion medium with 25 mM ethanol starting at t = 20 min. The result of this experiment revealed that ethanol induced a significant suppression of both the basal glucose release and the lactate-induced glucose release (**Figure 6**, EtOH).

## Discussion

In this study, we have devised conditions for delivery of a therapeutic concentration of metformin to the perfused rat liver, and we have investigated the acute effects of metformin on glucose release. To estimate the concentration and distribution of metformin we have used MALDI-MSI, which revealed that the drug can be detected at sub-micromolar concentration in the liver. We have discovered that metformin is accumulated from the perfusion medium into the liver with kinetic values indicating OCT1-mediated uptake (Wang et al., 2002). We found that metformin did not suppress the basal rate of glucose production, or glucose production forced by high lactate. This finding challenges the view that metformin causes an acute inhibition of glycerol 3-phosphate dehydrogenase (Madiraju et al., 2014), inducing a shift in the NADH/NAD ratio, thereby attenuating gluconeogenesis.

The complex chemical composition of the liver and its large number of endogenous low molecular weight metabolites renders the identification of peaks in the mass spectrum challenging. To unambiguously assign the peak of metformin in the mass spectrum of the liver tissue we used both the MS and MS/MS modalities. The resulting MS/MS mass spectrum showed a fragmentation pattern consistent with that derived for metformin based on its structure, in agreement with published observations (Michel et al., 2015). The 130.16 *m/z* peak was assigned to metformin, and there was no significant background from endogenous compounds in that region. This allowed for an estimation of the detection limit to <250 nM for metformin in the liver. This is well below the measured therapeutic metformin concentration in human plasma (Tucker et al., 1981; Christensen et al., 2011), as well as that in rodent tissues (Wilcock and Bailey, 1994), and in mouse xenograft tumors (Chandel et al., 2016; Dowling et al., 2016). This indicates that MALDI-MSI can be employed to determine the distribution of metformin in organ systems in general. Our results indicated that metformin was effectively accumulated into the liver parenchyma during the 5-min loading period. In the liver, uptake of metformin is mediated primarily by the OCT1 transporter with a K_m_ of 0.377 μM (Wang et al., 2002), which is in agreement with the uptake velocity derived from our MALDI-MSI measurements.

Quantification by mass spectrometry poses several challenges, and unique standard curves need be determined for each experimental setting, depending of the chemical background of the sample, the matrix deposition method, the ionization method, and the detector system. Orally or intravenously administered drugs reach the target tissue through the vascular system and become embedded within tissue matrix. This renders the quantification by external standards problematic due to the differences in ionization efficiency between embedded and external drug molecules. To solve these problems, we applied known quantities of a standard solution of metformin both under and on top of liver thin-sections. The results of the MALDI-MSI measurements indicated that applying the standard under the tissue thin-section gave a somewhat higher signal intensity than for standard applied on top of the thin-sections, probably because the former was located closer to the conducting surface of the glass slide, hence facilitating the ionization. To mimic the situation where the drug is embedded within the tissue matrix we used the average values obtained from the standard under and standard on top measurements. Alternative quantification methods employing tissue homogenates have been described (Jadoul et al., 2015), which are promising, but will require further scrutiny to ascertain the chemical integrity of the tissue.

**FIGURE 7:**
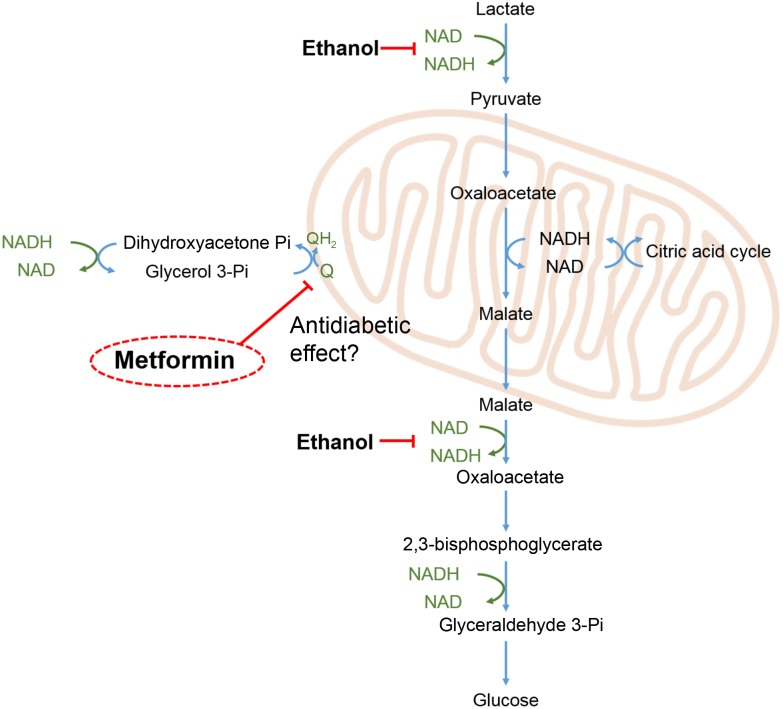
Redox shuttles during gluconeogenesis. During gluconeogenesis from lactate, e.g., during exercise, pyridine nucleotides undergo continuous redox cycling to support the redox reactions of the involved pathways. Imbalances in the redox balance of the pyridine nucleotide couples (marked in green), are adjusted by redox shuttle pathways including the glycerol 3-phosphate shunt, feeding excess electrons into the mitochondrial respiratory chain leading to reduction of oxygen. Ethanol oxidation to acetic acid by alcohol dehydrogenase and aldehyde dehydrogenase leads to a build-up of NADH and thereby an inhibition of glucose release as indicated in the scheme. Metformin has been proposed to exert its antidiabetic effect by inhibiting mitochondrial glycerol 3-phosphate dehydrogenase (EC 1.1.5.3) (Madiraju et al., 2014), as indicated in the scheme.

To investigate the acute metabolic effects of metformin we devised an experimental protocol to measure glucose release both at its basal level and when forced by high lactate. By performing the transition from basal to lactate-induced glucose release two times during each perfusion, without and with metformin, each liver served as its own control. This protocol reduced variations in glucose release arising from differences in the size of the liver lobe and from individual differences in the liver metabolism between the animals. During gluconeogenesis from lactate several reaction pathways are operating to maintain the equilibrium in the redox state of the NADP/NAD and NADPH/NADP couples, as shown in **Figure 7**. Most importantly the glycerol 3-phosphate shuttle, resulting in the oxidation of NADH and the transfer of its electrons to the mitochondrial respiratory chain, is required for the continuous conversion of lactate to glucose. It has been proposed that the antidiabetic effect of metformin is due to an inhibition of the enzyme glycerol 3-phosphate dehydrogenase leading to an accumulation of NADH, and hence to a diminished conversion of lactate to glucose (Madiraju et al., 2014). Our results do not lend support to this mechanism since we did not observe any significant suppression neither of the basal glucose release nor of the lactate-induced glucose release. In contrast, supplementing the medium with ethanol instead of metformin had an immediate inhibitory effect on the glucose release, consistent with an accumulation of reduced pyridine nucleotides caused by the oxidation of ethanol into acetic acid.

Our findings are in line with human studies showing that metformin does not exert any acute suppressive effect on glucose release from the liver when administered intravenously (Sum et al., 1992) or orally (Widén et al., 1992). Collectively, these human studies and our organ perfusion data suggest that metformin, at a therapeutically relevant low 10^-5^ M concentration, does not affect glucose release through direct inhibition of some target enzyme(s). Instead, the time lag required for metformin to take effect, points to the necessity of signal transduction events and transcriptional activation to occur, in consequence, leading to a gradual remodeling of the metabolic web, requiring days to reach maximal effect (Bailey, 1992). Therefore, it is likely that the anti-diabetic effect of metformin to a major extent is mediated through molecular mechanisms that require transcriptional regulation, leading to a reprogramming of the metabolic network. This is supported by the finding that common gene variants, which to the largest extent affect the response to metformin, are metformin transporters and transcription factors, such as HNR1A, HNF4A, PPARgC1A, MEF2A, MEF2D (Jablonski et al., 2010), and not enzymes of the metabolic pathways.

Our study shows that the metformin concentration can estimated by MALDI-MSI in rat liver, with a maximum sensitivity below the therapeutic concentration range. Therefore, MALDI-MSI will likely become a valuable tool to investigate the penetration and distribution of metformin into other solid tissues in rodents and humans. The MALDI-MSI technique may assist in providing essential data in future studies of metformin in tumor and neurodegenerative therapies.

## Author Contributions

OE and ML: planned and supervised the project; GC, EN, MM, AC, RS, ML, and OE: performed the experiments; OE and GC: wrote the manuscript; MB, EB, DL, and ML: revised the manuscript critically for important intellectual content. All co-authors approved the final version of the manuscript.

## Conflict of Interest Statement

EB was employed by company Tethis S.p.A. The other authors declare that the research was conducted in the absence of any commercial or financial relationships that could be construed as a potential conflict of interest.

## Footnotes

1 http://scils.de/

## Abbreviations

HCCA: α-cyano 4-hydrocinnamic acid
MALDI-MSI: matrix-assisted laser desorption/ionization mass spectrometry imaging
T2D: type 2 diabetes
TOF: time-of-flight
